# Network evolution of regional brain volumes in young children reflects neurocognitive scores and mother’s education

**DOI:** 10.1038/s41598-023-29797-1

**Published:** 2023-02-20

**Authors:** Yidong Zhou, Hans-Georg Müller, Changbo Zhu, Yaqing Chen, Jane-Ling Wang, Jonathan O’Muircheartaigh, Muriel Bruchhage, Sean Deoni, RESONANCE Consortium, Joseph Braun, Muriel Bruchhage, Susan Carnell, Sean Deoni, Viren D’Sa, Matthew Huentelman, Vanja Klepac-Ceraj, Monique LeBourgeois, Hans-Georg Müller, Jonathan O’Muircheartaigh, Jane-Ling Wang

**Affiliations:** 1 Department of Statistics, University of California, Davis, Davis, CA 95616 USA; 2 Department of Applied and Computational Mathematics and Statistics, University of Notre Dame, Notre Dame, IN 46556 USA; 3 Department of Statistics, Rutgers University, New Brunswick, NJ 08901 USA; 4 Centre for the Developing Brain, School of Biomedical Engineering and Imaging Sciences, King’s College London, London, UK; 5 Department of Forensic and Neurodevelopmental Sciences, Institute of Psychiatry, Psychology and Neuroscience, King’s College London, London, UK; 6 MRC Centre for Neurodevelopmental Disorders, King’s College London, London, UK; 7 Department of Pediatrics, Warren Alpert Medical School at Brown University, Providence, USA; 8 Department of Diagnostic Imaging, Rhode Island Hospital, Providence, USA; 9 Institute of Social Sciences, Stavanger University, Stavanger, 4021 Norway; 10 Maternal, Newborn, and Child Health Discovery and Tools, Bill and Melinda Gates Foundation, Seattle, WA USA; 11 Brown University School of Public Health, Brown University, Providence, RI 02912 USA; 12 Department of Psychiatry and Behavioral Sciences, Johns Hopkins University, Baltimore, MD 21218 USA; 13 Department of Pediatrics, Warren Alpert Medical School at Brown University, Providence, RI 02912 USA; 14 Neurobehavioral Research Unit, Translational Genomics Research Institute, Phoenix, AZ 85004 USA; 15 Department of Biological Sciences, Wellesley College, Wellesley, MA 02481 USA; 16 Integrative Physiology, University of Colorado at Boulder, Boulder, CO 80309 USA

**Keywords:** Cognitive neuroscience, Learning and memory, Sexual behaviour, Social behaviour

## Abstract

The maturation of regional brain volumes from birth to preadolescence is a critical developmental process that underlies emerging brain structural connectivity and function. Regulated by genes and environment, the coordinated growth of different brain regions plays an important role in cognitive development. Current knowledge about structural network evolution is limited, partly due to the sparse and irregular nature of most longitudinal neuroimaging data. In particular, it is unknown how factors such as mother’s education or sex of the child impact the structural network evolution. To address this issue, we propose a method to construct evolving structural networks and study how the evolving connections among brain regions as reflected at the network level are related to maternal education and biological sex of the child and also how they are associated with cognitive development. Our methodology is based on applying local Fréchet regression to longitudinal neuroimaging data acquired from the RESONANCE cohort, a cohort of healthy children (245 females and 309 males) ranging in age from 9 weeks to 10 years. Our findings reveal that sustained highly coordinated volume growth across brain regions is associated with lower maternal education and lower cognitive development. This suggests that higher neurocognitive performance levels in children are associated with increased variability of regional growth patterns as children age.

## Introduction

The maturation of the human brain during early development requires coordinated growth of different brain regions over time. While the total brain volume is around $80\%$ of the adult volume at 2 years of age^1^, there are substantial differences across specific brain regions that mature at different speeds. Advances in neuroimaging techniques, including postprocessing procedures of regional parcellation, have made it possible to quantify the volumes of specific brain areas. Brain networks, i.e., networks with brain regions as nodes and regional connections as edges, provide a unique perspective in modeling and understanding the structure and functioning of brains using graph theoretical analysis approaches^2^. Various types of brain networks and specifically structural covariance networks (SCNs)^3,4^ were widely investigated using diffusion MRI^5^ or structural MRI data^6^. For the latter, brain regional connections are typically derived from correlations of cortical thickness^7–9^ or volume^10–12^.

Postnatal human brain development, especially from birth to the onset of adolescence, is increasingly recognized to play an important role in establishing life-long cognitive abilities^13^ and behaviors^14^. Furthermore, early maturational processes have been shown to be accompanied by functional changes of brain networks at rest^15^. While there is very little work investigating network features of structural brain evolution during early brain development, even fewer studies utilize longitudinal data. This could be partly due to data sparsity as a result of difficulties with image acquisition in very young children and limited methodology to construct networks that reflect the growth dynamics of brain regions. As a result, much of the existing literature has been focused on cross-sectional analyses, typically considering only a limited number of pre-specified age bins to study differences in brain structure with age^5,6,11^. Our study is motivated by the need for methodology to assess the specific impact of subject-specific factors on the evolution of SCNs and specifically to assess the impact of sex and maternal education. The latter are known to be associated with general brain and cognitive development, while their effect on SCN evolution has remained unknown. Previously, the SCN corresponding to a specific age bin was estimated by pooling data recorded within that age bin^9,10^, where potential bias may be introduced since actual ages of measurements were not utilized. Additionally, previous longitudinal studies suffered from small sample sizes that may lead to considerable random fluctuations.

There is a large literature concerning the influence of genes and environment on brain structure and function^16,17^. Sex differences in brain structure and function have been well documented at birth and in postnatal development^4^. Maternal education^18^, a central aspect of socioeconomic status^19^, has been shown to play an important role in language development, reading, and education attainment^20^. However, the factors that influence evolving SCNs during brain maturation have received little attention and are poorly understood. This study aims at filling this gap in current knowledge.

While previous studies have shown that early and prolonged changes in brain volume, shape, and growth are associated with cognitive development^13^, much remains unknown. While one study^9^ connected time-varying SCNs to a motor task response time, no previous studies assessed the relation of overall cognitive development, such as scores in the Mullen Scales of Early Learning^21^ with evolving structural brain networks. To further our understanding of the regulating mechanisms of brain maturation during childhood, it is vital to close the existing gaps in knowledge, especially how developing SCNs are associated with sex, maternal education, and cognitive development.

Recent neuroimaging studies have demonstrated that SCNs can be meaningfully characterized by modularity and global efficiency^3,4^. Modularity^22^ is a measure of network segregation. Larger values of modularity reflect high connectivity between nodes within the same modules and low connectivity between nodes that belong to different modules. Global efficiency^23^ is a scaled measure of how many steps it takes when moving through the network from one node to another, where higher efficiency means that on average fewer steps are needed. Modularity and global efficiency are widely used to characterize brain networks. These summary measures are especially useful to quantify time-varying changes in SCNs and thus can be used to assess the network evolution of brain volumes^9^.

We adopt here a methodology that was previously introduced in Petersen et al.^24^ and is aimed at obtaining time-varying structural covariance using local Fréchet regression^25,26^. This approach has been shown to lead to consistent estimation of the cross-sectional time-varying covariance structure, even in very sparse cases, where each subject is visited at only one random age and, specifically, when these ages are different for each subject, as is invariably the case in neurodevelopmental studies. The Fréchet estimation approach overcomes difficulties caused by the sparsity and irregularity of the data and makes it possible to obtain the structural covariance matrix at any age of interest.

In this paper, we construct time-varying SCNs for 554 children from 9 weeks to 10 years of age using brain volumes extracted from structural MRI data, and investigate the temporal evolution of these networks using modularity and global efficiency. We study here how the time co-evolution of brain volumes and the ensuing SCNs are related to the biological sex of the child and the education level of the mother, and also their association with cognitive development as represented by the Mullen ELC scores. Further details on demographics, MRI acquisition and analysis, network measures, dynamic brain structural covariance network modeling, local Fréchet regression^25,26^, inference using permutation tests, and statistical analyses are described in “Methods”.

## Results

### Sex and maternal education effects

**Figure 1:**
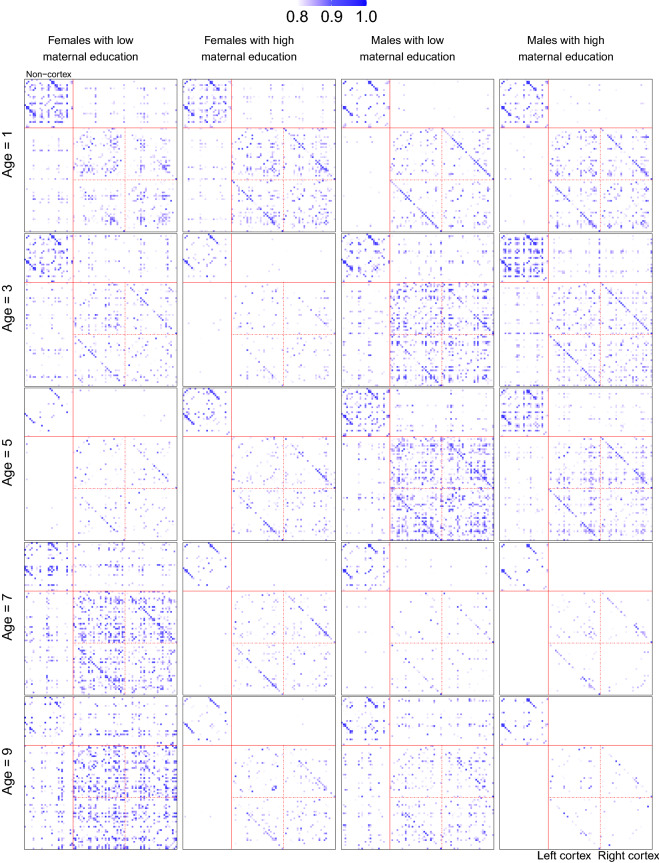
SCNs represented as heatmaps at ages 1, 3, 5, 7, 9 years for the four groups divided by sex and maternal education. Rows from top to bottom correspond to the five ages. Columns from left to right correspond to the four groups: females with low maternal education, females with high maternal education, males with low maternal education, males with high maternal education. The top left block contains the non-cortex regions, while the bottom right contains the left and right cortex regions; see supplementary material S1 for details of ROIs.

The total sample was divided into four groups using sex and maternal education to study their effects on the evolution of SCNs. The four groups are females with low maternal education, females with high maternal education, males with low maternal education, and males with high maternal education. For each group, the estimated SCNs at different ages were obtained using local Fréchet regression; see “Methods” for details. SCNs at ages 1, 3, 5, 7, 9 years using threshold $\theta =0.8$ are demonstrated as heatmaps in Fig. 1, where columns correspond to the four groups and within each column the SCNs are arranged according to age. See the supplementary material S1 for a circular visualization of the same SCNs.

We observe from Fig. 1 that the 91 ROIs (for details see supplementary material S1) separate with increasing age into two communities: a small community in top left and a large community in bottom right. This trend is more pronounced for children with a high maternal education. The two communities are found to coincide with the non-cortical and the cortical area of the brain, respectively. The cortical area consists of 31 regions from the left hemisphere and 31 regions from the right hemisphere.

To assess the statistical significance of sex and maternal education effects on the evolution of SCNs, permutation tests were applied at ages $1,2, \dots , 9$ years, using modularity and global efficiency with $Q=5000$. The testing procedure is described in “Methods” and corresponding *p*-values are reported in the first two columns of Table 1. Recall that the test statistic is defined as the integral of average network measure over age $t_j$ to $t_N$ (specifically, $t_N=9$ in our analysis) while conducting permutation tests at age $t_j$. We are thus essentially testing whether the sex and maternal education effects are significant starting age $t_j$, rather than solely at age $t_j$. We observe that the sex and maternal education effects are significant with $p<0.05$ with respect to modularity at the later childhood period (age 6, 7, 8, 9 years). Specifically, for ages 7 and 9, the effects are significant with $p<0.01$. However, none of the tests are significant with respect to global efficiency. Modularity is a measure of network segregation, while global efficiency in contrast is a measure of network integration. This suggests that sex and maternal education shape the evolution of SCNs more on their segregation than on their integration aspects.

**Table 1:** *p*-values for the permutation tests assessing the sex and maternal education effects and cognitive development association with the evolution of brain region volumes based on modularity and global efficiency, where *p*-values less than 0.05 are emphasized in bold.

Age	Sex and maternal education		Cognitive development	
	Modularity	Global efficiency	Modularity	Global efficiency
1	0.072	0.434	0.067	**0.035**
2	0.051	0.440	0.056	**0.030**
3	0.084	0.467	0.055	**0.028**
4	0.063	0.545	0.107	**0.050**
5	0.063	0.541	0.142	**0.042**
6	**0.013**	0.200	0.138	**0.020**
7	**0.008**	0.088	0.087	**0.003**
8	**0.012**	0.215	0.099	**0.004**
9	**0.009**	0.430	0.107	**0.003**

**Figure 2:**
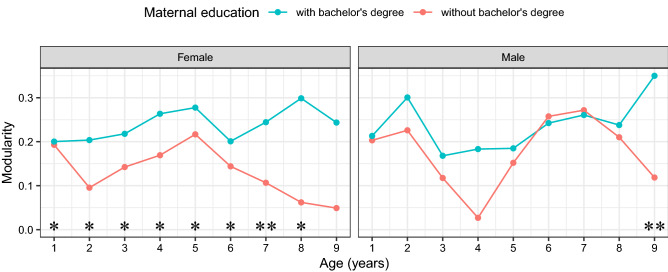
Modularity of SCNs at ages 1–9 for the four groups divided by sex and maternal education. Significance of differences is indicated separately for each comparison (*0.05, **0.01).

To better understand the effects of sex and maternal education on the evolution of SCNs, a plot of modularity for the four groups at the nine ages is shown in Fig. 2. It can be seen that the effect of maternal education on the evolution of SCNs is much more pronounced than that of sex with respect to modularity. As a complementary analysis, we assessed the significance of maternal education effects for females and males separately, using permutation tests, with *p*-values reported in Table 2. We found that significance with respect to modularity is mainly due to the data collected for the  females, which suggests that maternal education shapes the evolution of SCNs for females more than for males.

**Table 2:** *p*-values for the permutation tests assessing the maternal education effects on the evolution of brain region volumes within females and males based on modularity and global efficiency, where *p*-values less than 0.05 are emphasized in bold.

Age	Maternal education within females		Maternal education within males	
	Modularity	Global efficiency	Modularity	Global efficiency
1	**0.024**	0.112	0.342	0.702
2	**0.017**	0.132	0.309	0.662
3	**0.024**	0.181	0.337	0.636
4	**0.018**	0.218	0.320	0.563
5	**0.019**	0.174	0.520	0.793
6	**0.011**	0.080	0.510	0.838
7	**0.008**	**0.039**	0.355	0.689
8	**0.014**	0.079	0.188	0.637
9	0.081	0.358	**0.034**	0.246

### Cognitive development association

**Figure 3:**
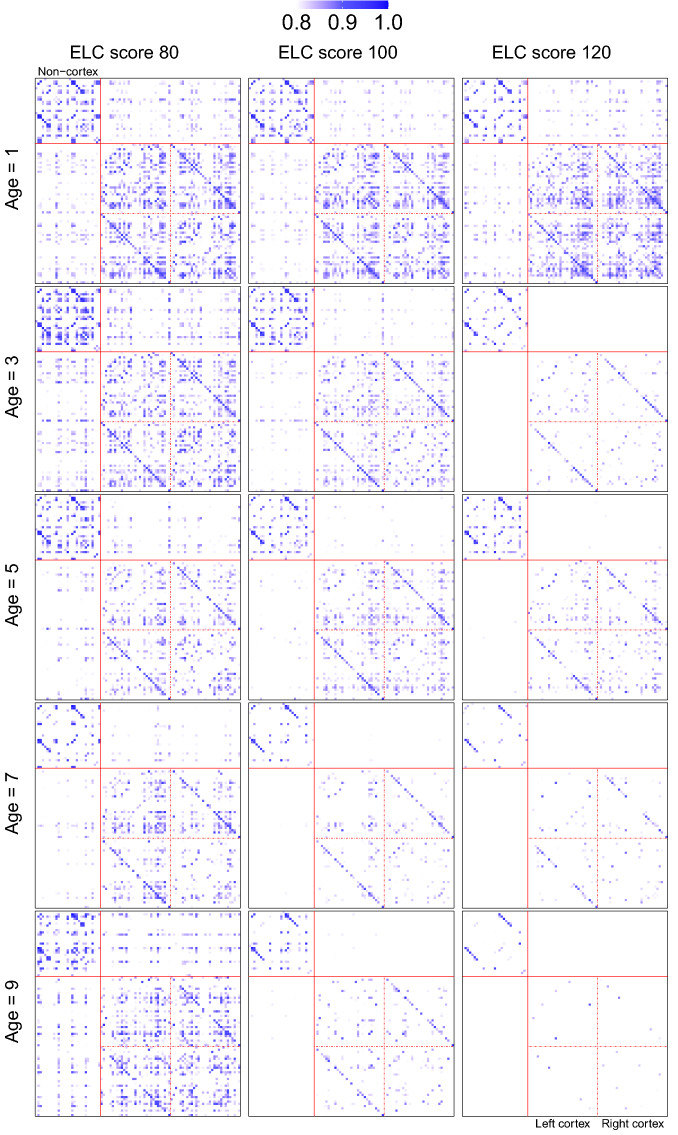
SCNs represented as heatmaps at ages 1, 3, 5, 7, 9 years for ELC scores 80, 100, and 120. Rows from top to bottom correspond to the five selected ages. Columns from left to right correspond to the three ELC scores. The top left block contains the non-cortex regions, while the bottom right contains the left and right cortex regions; see supplementary material S1 for details of ROIs.

We studied the association between cognitive development and the evolution of SCNs through local Fréchet regression, where age and ELC score were incorporated as covariates and SCN as the responses. We estimated the time-varying SCNs at three different levels of cognitive development (ELC scores 80, 100, 120), where for each level ages 1, 2, . . . , 9 years are considered. SCNs at ages 1, 3, 5, 7, 9 years using threshold $\theta = 0.8$ are demonstrated as heatmaps in Fig. 3, where each row contains three SCNs corresponding to three different ELC scores and each column contains five SCNs at the five highlighted ages. See the supplementary material S1 for a circular visualization of the same SCNs. We observe from Fig. 3 that highly correlated volumes are associated with lower Mullen scores, especially at later ages.

Similarly as for sex and maternal education, permutation tests were performed to assess the significance of the association between cognitive development and the evolution of SCNs. Specifically, the original sample was permutated $Q=5000$ times and the permutation test as described in “Methods” was conducted at ages 1–9, one year apart. The *p*-values are reported in the last two columns of Table 1. It can be seen that the association between cognitive development and the evolution of SCNs is significant with respect to global efficiency at the level of 0.05 at ages from 1 to 6 and at the level of 0.01 at ages from 7 to 9. However, none of the tests are significant with respect to modularity, which suggests that the association between cognitive development and the evolution of SCNs is mainly an association with network integration.

**Figure 4:**
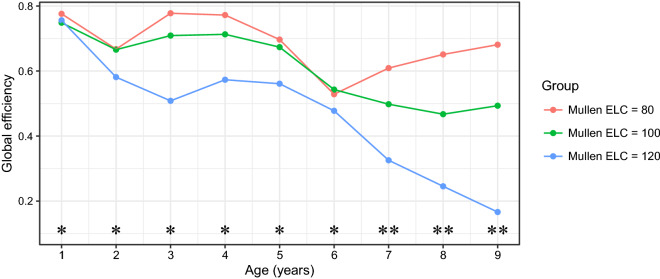
Global efficiency of SCNs at ages 1–9 with one year apart for three different ELC scores 80, 100, 120. Significance of differences is indicated separately for each comparison (*0.05, **0.01).

Global efficiency of the time-varying SCNs for the three ELC scores is shown in Fig. 4. It suggests that lower level of cognitive development is associated with higher global efficiency, implying greater network integration. More importantly, the three curves in the plot are almost perfectly separated, indicating significant association between cognitive development and the evolution of SCNs.

## Discussion

In this study, we investigated how evolving SCNs are associated with biological sex, maternal education and cognitive development (ELC score). Highly coordinated brain volume growth was found to be associated with lower maternal education as well as with lower level of cognitive development. These differences were particularly present in older children at ages 7–9, and at early ages for cognitive development. Our results indicate that biological sex and maternal education shape the evolution of SCNs more on their segregation, while cognitive development as measured by the ELC scores is primarily associated with network integration. While sex and maternal education have significant effects at ages 6–9, the association between cognitive development and the evolution of SCNs is significant throughout childhood (ages 1–9). The effect of maternal education on the evolution of SCNs was shown to be more pronounced than that of sex with respect to modularity. In addition, follow-up permutation tests revealed that maternal education shapes the evolution of SCNs for females more than for males.

The impact of sex and maternal education on the evolution of SCNs can be quantified by modularity. More modular networks are thought to specialize in information processing^27^, to perform focal functions^28^ and to support complex neural dynamics^29^, suggesting more specialized functions of these networks. In Fig. 2, we observe that at high maternal education levels, network modularity increases for males across ages, with some fluctuations at early and later ages, while the effect flattens out after age 5 for females. For the case of low maternal education, network modularity decreases after age 6 for both females and males. For males, we observe that the modularity of SCNs follows an inverted U-shaped curve during infancy, with the modularity of 2-year-olds being higher than that of 1-year-olds and 3-year-olds. This descriptive pattern lends support to previous findings in Fan et al.^10^ that the strength of network segregation of SCNs during infancy peaks at age 2.

After age 3, network modularity tends to increase until age 7, followed by a decrease, which is in line with previous work^7^ reporting that the strength of network segregation reaches its highest values at 6–7 years of age. This might be related to the start of schooling. Females display similar patterns but with a two-year lag ahead, which is in line with previous findings that females’ brains integrate and shape themselves two or three years earlier than those of males^30^. Compared with low maternal education, high maternal education is associated with higher modularity and exhibits a slightly different pattern over the last two years. The effect of maternal education seems to differ between males and females, mostly manifested in the low maternal education category after age 5. The supplementary analysis S1 as shown in Table 2 suggests that maternal education has a greater impact on the evolution of SCNs for females than males in terms of modularity. In a previous study^31^, the authors showed that lower SES is associated with worse behavioral inhibition over time and a concurrent increase in anterior cingulate activation, but only in females. Additionally, evidence shows that SES interacts with sex on mental health, where females benefit more from higher SES in terms of improved mental health^32^.

Permutation tests revealed that the association between cognitive development and the evolution of SCNs is reflected by network integration, which was shown to remain significant throughout the age range, especially in later childhood. Particularly, lower levels of cognitive functioning were associated with higher global efficiency, implying greater network integration. For children with low and average ELC scores, global efficiency displayed U-shaped curves during infancy, with the global efficiency of 2-year-olds being lower than that of 1-year-olds and 3-year-olds. Up to age five, SCNs for children with low and average ELC scores showed similar global efficiency. The contrast in global efficiency among all three groups increased sharply at ages seven and beyond, reflecting accelerating divergence in brain structure among the three groups after age seven.

The gap in global efficiency between the three groups with different ELC scores is increased in the age period of 3-5, when brain growth markedly slows and in the age period 7-9, overlapping in time with schooling. Importantly, Fig. 4 indicates a decrease in global efficiency in the coordination and thus overall cognitive function with age for children with high cognitive development. Thus, increased path length (decreased global efficiency) could indicate the importance of long-range connections in the brain. In the mammalian brain, the distance between cortical regions largely determines the extent to which they are inter-connected^33,34^, and higher-order cognition is mirrored and implemented via distributed cortical networks that are linked via long-range connections in the human brain^35^. However, long-range connections are biologically expensive, and it is unknown how the computational advantages that long-range connections provide overcome the associated wiring costs, with sensory/motor networks showing locally clustered connectivity profiles, while more complex transmodal association cortices show long-range connections^36,37^. While our results do not directly pertain to wiring costs, but rather to the tightness of the orchestration of longitudinal growth across brain regions, it is possible that the tight coordination that persists in the presence of lower neurocognitive performance is characteristic for early brain growth and may indicate less potential for brain differentiation and associated performance. Such considerations could indicate that at higher levels of cognitive ability (i.e., a standardized ELC score of 120) more long-range connections are present.

The present study benefits from a number of strengths. The Fréchet regression approach makes it possible to estimate the structural covariance at any age of interest, rather than at certain pre-specified age bins, which overcomes difficulties caused by the sparsity and irregularity of the data. The longitudinal design and large sample size allow for more powerful and accurate tests of determinants of brain maturation. Additionally, the present study covers a wide range of ages, from 9 weeks to 10 years, supporting the understanding of brain maturation during childhood. Several limitations must also be considered. First, we only studied the impact of sex and maternal education on brain maturation at the network level. Further research could be conducted to determine the precise mechanism by which sex and maternal education mediate brain maturation at the ROI or neuronal level. Second, the current study focused on tests based on network measures, rather than the network itself. With adequate data, one may construct SCNs using the across-subject correlations of regional volumes and employ a network-response regression approach^38^ to uncover determinants of brain maturation.

In conclusion, we present a study of the maturation of regional brain volumes using time-varying structural brain networks and demonstrate the utility of local Fréchet regression for the estimation of time-varying structural covariance from sparse and irregular longitudinal neuroimaging data. The developmental pattern of the corresponding brain networks contributes to a better understanding of the maturation of the human brain and how it interacts with biological sex and maternal education.

## Methods

### Subjects details and demographics

**Figure 5:**
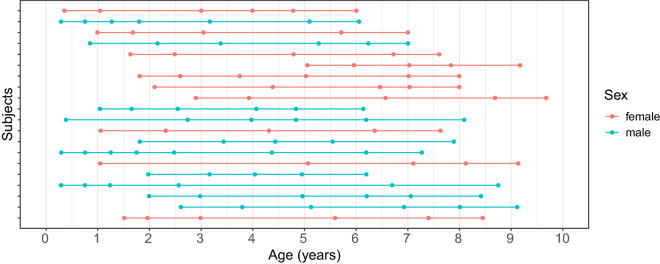
A longitudinal event plot demonstrating the distribution of ages of visits per child for the 10 females and 10 males with the most visits. Each row corresponds to a child, where dots denote the event times where visits took place.

**Figure 6:**
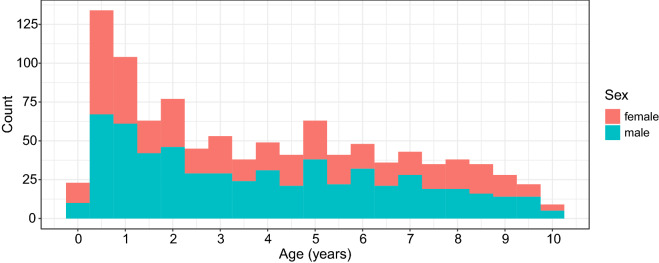
Histogram for ages at time of visits for the RESONANCE cohort.

We included 554 children (245 females and 309 males) in this study, ranging from 9 weeks to 10 years of age. All data were drawn from an ongoing RESONANCE longitudinal study of healthy and neurotypical brain and cognitive development from early childhood to preadolescence, based at Brown University in Providence, RI, USA. RESONANCE^39,40^ was designed as an accelerated longitudinal study of a large community cohort of healthy children, with around half of the cohort enrolled between the ages of 2 and 8 months, and the remaining children between the ages of two and four years. Children in this study are typically enrolled between birth and 2 years of age, and then followed with repeated study visits and assessments at 6 or 12-month increments depending on child age. During each visit, multi-modal MRI, performance and parent-reported measures of cognitive and behavioral functioning, anthropometry, and biospecimen collection were collected. For our analysis, data from 1,025 visits were included. Table 3 shows the number of visits per child, where one can see that most children had completed only a single visit. The distribution of ages of visits per child for the 10 females and 10 males with the most visits is illustrated in Fig. 5, which exemplifies the irregular design of the study. The histogram for ages of visits for all children is provided in Fig. 6.

**Table 3:** Distribution of numbers of visits per child.

Number of visits per child	1	2	3	4	5	6	7	8	Total number of visits
Females	142	60	14	15	11	3	0	0	437
Males	163	71	42	19	6	6	1	1	588

Children with known significant risk factors for developmental abnormalities or cognitive impairments were excluded from enrollment. Exclusion criteria included: born prematurely (37 weeks) or small for gestational age (1500 g), self-reported in utero exposure to alcohol, cigarette smoke, or illicit substances; fetal ultrasound abnormalities; complicated delivery resulting in 5-minute APGAR scores of 8 and/or NCU admission; neurological pediatric disorder (e.g., head injury resulting in loss of consciousness, epilepsy); and known psychiatric or learning disorder in the infant, parents, or siblings (including maternal depression requiring medication in the year prior to pregnancy).

### Ethics statement

The host institutions, Brown University and Lifespan Institutional Review Boards, provided ethical approval for this study. All study-related procedures were performed in accordance with the research ethics guidelines outlined in the Declaration of Helsinki. Written informed consent was acquired from all children’s parents or legal guardians.

### MRI acquisition and analysis

For all MR image acquisition, children under 4 years of age were scanned during natural and non-sedated sleep and older children were imaged whilst watching a movie or other video. Our imaging protocol included relaxometry, multi-shell diffusion, resting-state connectivity, and magnetic resonance spectroscopy acquisitions in addition to the anatomical data. As a result, depending on child compliance (sleeping and/or motion), high quality anatomical data were not collected or available for every child at every scan time-point. Following data acquisition, scans were inspected to ensure there were no motion-related artifacts and image blurring and ghosting. T1-weighted anatomical data were acquired on a 3T Siemens Trio scanner with a 12-channel head RF array. T1-weighted magnetization-prepared rapid acquisition gradient echo anatomical data were acquired with an isotropic voxel volume of $1.2 \times 1.2 \times 1.2 {\text{ mm}}^3$, resampled to $0.9 \times 0.9 \times 0.9 {\text{ mm}}^3$. Sequence specific parameters were: TE = 6.9 ms; TR = 16 ms; inversion preparation time = 950 ms; flip angle = 15 degrees; BW = 450 Hz/Pixel. The acquisition matrix and field of view were varied according to child head size in order to maintain a constant voxel volume and spatial resolution across all ages^41^. Using a multistep registration procedure^42^, a series of age-specific anatomical T1-weighted templates were created corresponding to 3, 6, 9, 12, 15, 18, 21, 24, 30, 36, 42, 48, 60, 72, 84, 96 and 108-month ages. At least 10 females and 10 males were included in each template. An overall study template was then created from these age templates, which was aligned to the MNI152 template^43^. Each child’s anatomical T1-weighted image was transformed into MNI space by first aligning to their age-appropriate template and then applying the pre-computed transformation to MNI space, with the calculated individual forward and reverse transformations saved and used for the volumetric analysis described below. All template creation and image alignment were performed using a 3D nonlinear approach^44^ with cross-correlation and mutual information cost functions. We then applied the Desikan-Killiany-Tourville (DKT) cortical labeling protocol, FreeSurfer’s wmparc and aseg non-cortical (plus white matter) labels through Mindboggle^45,46^, resulting in volumetric output from 96 brain regions. Five regions with very small volumes were excluded: left inferior lateral ventricle, left vessel, right inferior lateral ventricle, left basal forebrain, and right basal forebrain.

### Socioeconomic status and neurocognitive assessments

Alongside neuroimaging data, maternal education and cognitive development were also assessed for each child. Maternal education^18^, as an important component of family socioeconomic status^19^, was assigned a numerical value based on the education level of the mother. These values were as follows: less than seventh grade = 1; junior high school = 2; partial high school = 3; high school graduate = 4; partial college or specialized training = 5; college graduate = 6; graduate training = 7. In our analysis, maternal education was transformed to a categorical variable by setting values less than 6 to low and high otherwise. Then low maternal education corresponds to a mother without bachelor’s degree and high corresponds to a mother with bachelor’s degree. The cognitive development was assessed using a combination of observed performance and parent-reported measures, where Early Learning Composite (ELC), a composite score combining overall visual, motor, and language functioning from the Mullen Scales of Early Learning^21^, was used to assess overall cognitive functioning of each child. For the RESONANCE cohort, $60.26\%$ of the females and $63.76\%$ of the males have a mother with a bachelor’s degree. The mean and standard deviation of the ELC scores for females and males are 101.53 (15.56) and 97.23 (17.04), respectively, with standard deviation in parentheses.

### Network measures

We constructed simple, weighted, and undirected networks $G=(V, A)$ with a set of nodes $V=\{v_1, v_2, \dots , v_m\}$ and the associated adjacency matrix $A=\{a_{ij}\}_{i, j=1}^m$, indicating nodes $v_i$ and $v_j$ are either connected by an edge of weight $a_{ij}>0$, or else unconnected if $a_{ij}=0$. The strength of the network for segregation can be quantified by modularity^9,22^, a measure of the degree to which the network can be subdivided into clearly delineated and nonoverlapping groups. Higher modularity values represent stronger network segregation. Defining $s=\sum _{i, j=1}^ma_{ij}$ as the sum of all weights in *G*, $d_i=\sum _{j=1}^ma_{ij}$ as the weighted degree of node $v_i$ and $m_i$ as the module containing node $v_i$, the modularity of the network *G*^47^ is

1 $$\begin{aligned} Q=\frac{1}{s}\sum _{i, j=1}^m\left( a_{ij}-\frac{d_id_j}{s}\right) \delta _{m_i, m_j}, \end{aligned}$$

where $\delta _{m_i, m_j}=1$ if $m_i=m_j$ and 0 otherwise. In contrast to modularity, global efficiency^9,23^, a measure of the ability to rapidly combine specialized information from distributed brain regions, can be used to quantify the strength of integration of *G*. Global efficiency is defined as the average inverse of the weighted shortest path length of each node to all other nodes,

2 $$\begin{aligned} E=\frac{1}{m}\sum _{i=1}^m\frac{\sum _{j\ne i}l_{ij}^{-1}}{m-1}, \end{aligned}$$

where $l_{ij}$ is the weighted shortest path length between nodes $v_i$ and $v_j$, which is $l_{ij}=\sum _{a_{uv}\in g_{i\rightarrow j}}a_{uv}$, with $g_{i\rightarrow j}$ the shortest path (geodesic) between nodes $v_i$ and $v_j$. Note that $l_{ij}=\infty$ if nodes $v_i$ and $v_j$ are disconnected. Higher global efficiency is indicative of faster information transfer, or equivalently, of greater network integration.

### Local Fréchet regression for covariance matrices

To obtain age-specific structural covariance for brain regions, we require a regression model with age as covariate and structural covariance as the response. Since the space where covariance matrices reside is not a vector space, classical regression models do not apply due to the non-Euclidean nature of these objects. Local Fréchet regression^25,26^ is a nonparametric regression method for responses lying in metric spaces that are coupled with Euclidean covariates; it is well suited for our purposes.

Suppose $(T, C)\sim F$ is a random pair, where the covariate *T* takes values in $\mathbb {R}$, the response *C* is a random covariance matrix taking values in $\mathscr {S}_m$, the space of symmetric non-negative definite matrices of dimension *m*, and *F* is the joint distribution of (*T*, *C*) on $\mathbb {R}\times \mathscr {S}_m$. The conditional Fréchet mean, which corresponds to the regression function of *C* given $T=t$, is

3 $$\begin{aligned} \Sigma (t)=\mathop {\textrm{argmin}}_{\omega \in \mathscr {S}_m}M(\omega , t), \quad M(\cdot , t)=E\{d_F^2(C, \cdot )|T=t\}, \end{aligned}$$

where $d_F$ is the Frobenius metric defined as

$$\begin{aligned} d_F(\Sigma _1, \Sigma _2)=\Vert \Sigma _1-\Sigma _2\Vert _F=\{\textrm{trace}((\Sigma _1-\Sigma _2)'(\Sigma _1-\Sigma _2))\}^{1/2}, \end{aligned}$$

for two elements $\Sigma _1, \Sigma _2\in \mathscr {S}_m$ with the single quote denoting the transpose of a matrix.

Further suppose that $\{(T_i, C_i)\}_{i=1}^n$ are independent realizations of (*T*, *C*). Local Fréchet regression aims to estimate the conditional Fréchet mean $\Sigma (t)$ as per (3) for $t\in \mathbb {R}$ by

4 $$\begin{aligned} \hat{\Sigma }(t)=\mathop {\textrm{argmin}}_{C\in \mathscr {S}_m}\sum _{i=1}^ns_{iL}(t, h)d_F^2(C, C_i). \end{aligned}$$

Here $s_{iL}(t, h)=\frac{1}{\hat{\sigma }_0^2}K_h(T_i-t)[\hat{b}_2-\hat{b}_1(T_i-t)]$, where $\hat{b}_j=n^{-1}\sum _{i=1}^nK_h(T_i-t)(T_i-t)^j$ for $j=0, 1, 2$, $\hat{\sigma }_0^2=\hat{b}_0\hat{b}_2-\hat{b}_1^2$, and $K_h(\cdot )=h^{-1}K(\cdot /h)$ with $K(\cdot )$ a smoothing kernel and *h* a bandwidth.

### Dynamic brain structural covariance network modeling

We apply local Fréchet regression^25,26^ for the case where the responses are raw covariance matrices^24^ to obtain estimates for the SCN at a specific age. Denote the age domain $[0, T]\subset \mathbb {R}$ by $\mathscr {I}$ and the observed data by i.i.d. pairs $\{(T_i, Y_i)\}_{i=1}^n$, where $T_i\in \mathscr {I}$ represents the age at which the child was examined and $Y_i\in \mathbb {R}^m$ is a vector containing the brain region volumetric measurements for $m = 91$ brain regions of interest (ROIs) extracted from an MR image obtained during visits. For details of ROIs see supplementary material S1. Suppose the random pair (*T*, *Y*) taking values in $\mathscr {I}\times \mathbb {R}^m$ follows the same joint distribution as $(T_i, Y_i)$ for all *i*. We aim to estimate the time-varying structural covariance $\Sigma (t)=\textrm{Cov}(Y|T=t)$, which has been implemented by means of local Fréchet regression in^24^. Specifically, the starting point are raw covariance matrices given by

$$\begin{aligned} \hat{C}_i = (Y_i-\hat{\mu }(T_i))(Y_i-\hat{\mu }(T_i))', \end{aligned}$$

where $\hat{\mu }(\cdot )$ is the estimate of the conditional mean function $\mu (\cdot )=E(Y|T=\cdot )$. Estimates of each component function $\mu _j(\cdot )$ of $\mu (\cdot ) = [\mu _1(\cdot ),\dots ,\mu _m(\cdot )]'$ can be obtained in a variety of ways, including smoothing splines and local polynomial methods^48^ or other scatterplot smoothers.

Estimates of the time-varying structural covariance $\Sigma (t)$ are obtained by taking $T_i$ as covariates coupled with $\hat{C}_i$ as responses in the local Fréchet regression step as per (4),

5 $$\begin{aligned} \hat{\Sigma }(t)=\mathop {\textrm{argmin}}_{C\in \mathscr {S}_m}\sum _{i=1}^ns_{iL}(t, h)d_F^2(C, \hat{C}_i). \end{aligned}$$

Estimates of time-varying structural correlations that quantify the dynamic correlations of the 91 brain region volumes $\hat{R}(t)$ are then obtained by standardizing $\hat{\Sigma }(t)$, i.e.,

6 $$\begin{aligned} \hat{R}(t)=[\textrm{diag}(\hat{\Sigma }(t))]^{-1/2}\hat{\Sigma }(t)[\textrm{diag}(\hat{\Sigma }(t))]^{-1/2}. \end{aligned}$$

Finally, weight-based thresholding^49^ is applied to $\hat{R}(t)=\{\hat{r}_{ij}(t)\}_{i, j=1}^m$ by setting diagonal entries to 0 and retaining only connections surpassing a threshold $\theta$. The resulting so-called adjacency matrices $\hat{A}(t)=\{\hat{a}_{ij}(t)\}_{i, j=1}^m$ uniquely characterize the SCNs at age *t*. Specifically, $\hat{a}_{ii}(t)=0$ for $1\le i\le m$ and

7 $$\begin{aligned} \hat{a}_{ij}(t)={\left\{ \begin{array}{ll}\hat{r}_{ij}(t),&{}\hat{r}_{ij}(t)\ge \theta ,\\ 0,&{}\hat{r}_{ij}(t)<\theta ,\end{array}\right. } \end{aligned}$$

for $1\le i\ne j\le m$. The above estimation procedure essentially enables us to estimate the SCN for any age between 0 an *T*. For ease of analysis and presentation, one can choose some representative ages as needed.

Each SCN may be characterized using a variety of network measures^47^, among which modularity and global efficiency as per (1) and (2) are widely used in the literature^9,50^. Since the choice of threshold $\theta$ is somewhat arbitrary, a range of thresholds is often analyzed to examine the $\theta$-sensitivity of selected network measures. More principled approaches include functional data analysis across a range of thresholds^51^, as well as integration of a given network measure across a range of thresholds, yielding the area under the curve (AUC), and statistical inference is then performed on the AUC^52–55^. The AUC represents a single summary measure across a range of thresholds, which makes it possible to characterize various properties of SCNs independent of the choice of a specific threshold. In our analysis, thresholds varying from 0.6 to 0.9 (7 values, 0.05 increments) were examined, where the lower and upper bounds 0.6 and 0.9 were chosen to enforce sparsity and avoid fragmentation of the resulting SCNs.

### Biological sex- and environment-regulated brain maturation

To investigate how sex (female or male) and maternal education (low or high) affect the evolution of brain region volumes, we divided the total sample into four groups using the above two categorical covariates (or determinants), corresponding to females with low maternal education, females with high maternal education, males with low maternal education and males with high maternal education, respectively. For each group, local Fréchet regression was applied and the predicted correlation matrices $\hat{R}(t)$ are obtained as in (6), which are then converted into SCNs for subsequent network visualization and network measure calculation.

Another primary focus of interest is the association between cognitive development and the evolution of brain region volumes. By including age and ELC score as covariates (determinants) in local Fréchet regression^25,26^, we are able to estimate the time-varying SCNs for different ELC scores, corresponding to different levels of cognitive development. Denote by $\textbf{X}_i=(T_i, Z_i)'$ the two covariates, age and ELC score, for the *i*th child. The estimation of structural covariance $\Sigma (\textbf{x})$ for $\textbf{x}=(t, z)'$ can be similarly obtained as in (5), i.e.,

8 $$\begin{aligned} \hat{\Sigma}(\textbf{x})=\mathop {\textrm{argmin}}_{C\in \mathscr {S}_m}\sum _{i=1}^ns_{iL}(\textbf{x}, h)d_F^2(C, \hat{C}_i), \end{aligned}$$

where the weight function $s_{iL}(\cdot , h)$ takes a slightly different form. Specifically,

$$\begin{aligned} s_{iL}(\textbf{x}, h)=\frac{1}{\hat{b}_0-\hat{b}_1'\hat{b}_2^{-1}\hat{b}_1}K_h(\textbf{X}_i-\textbf{x})[1-\hat{b}_1'\hat{b}_2^{-1}(\textbf{X}_i-\textbf{x})], \end{aligned}$$

where $\hat{b}_0=n^{-1}\sum _{i=1}^nK_h(\textbf{X}_i-\textbf{x}), \hat{b}_1=n^{-1}\sum _{i=1}^nK_h(\textbf{X}_i-\textbf{x})(\textbf{X}_i-\textbf{x})$, and $\hat{b}_2=n^{-1}\sum _{i=1}^nK_h(\textbf{X}_i-\textbf{x})(\textbf{X}_i-\textbf{x})(\textbf{X}_i-\textbf{x})'$. The time-varying SCNs for a specific level of cognitive development are then constructed by fixing an ELC score and varying ages from 0 to *T* in the estimation, followed by normalization and thresholding as per (6) and (7). In particular, three different ELC scores 80, 100, 120 (corresponding approximately to the $10\%$, $50\%$, $90\%$ quantiles of the sample distribution of the ELC scores) corresponding to low, average, high levels of cognitive development were considered. For each level of cognitive development, the estimation of time-varying structural covariance was obtained as per (8) by varying *t* and fixing *z* as the corresponding ELC score. After normalization and thresholding, we were able to estimate the time-varying SCNs for the three levels of cognitive development, the changing patterns and group differences of which are of interest.

### Inference using permutation tests

A permutation test^56,57^ was used to assess the statistical significance of sex and maternal education effects on the evolution of SCNs, where the original sample was randomly permuted *Q* times. This approach was implemented as follows: For the *q*th permutation, $n_k$ observations are randomly assigned to the *k*th group for $k=1, 2, 3, 4$, where $n_k$ is the number of observations in the original sample that belong to the *k*th group. Next local Fréchet regression is applied to each group and the SCNs are constructed by normalization and thresholding at *N* different ages, say $0< t_1< t_2< \cdots< t_N < 10$.

As a result, four time-varying SCNs $\hat{A}_k^{(q)}(t_j)$ for $k=1, 2, 3, 4$ and $j=1, 2, \ldots , N$ are obtained corresponding to the four groups for each threshold, where $\hat{A}_k^{(q)}(t_j)$ denotes the adjacency matrix associated with the SCN for group *k* at age $t_j$. The average network measure of interest over a range of thresholds varying from 0.6 to 0.9 (7 values, 0.05 increments), denoted by $\bar{M}_k^{(q)}(t_j)$, is then computed based on the resulting SCNs $\hat{A}_k^{(q)}(t_j)$. For each $t_j$, we estimate the integral of average network measures from $t_j$ to $t_N$ by $I_k^{(q)}(t_j):=\sum _{l=j}^N\bar{M}_k^{(q)}(t_l)(t_l-t_{l-1})$ where $t_0=0$. A permutation test can be conducted based on $I_k^{(q)}(t_j)$ at each $t_j$.

The test statistic is then the variance of $I_k^{(q)}(t_j)$ for $k=1, 2, 3, 4$, denoted by

$$\begin{aligned} T_j^{(q)}=\frac{1}{3}\sum _{k=1}^4(I_k^{(q)}(t_j)-\bar{I}^{(q)}(t_j))^2,\quad \bar{I}^{(q)}(t_j)=\frac{1}{4}\sum _{k=1}^4I_k^{(q)}(t_j). \end{aligned}$$

Finally, the one-sided *p*-value for the permutation test at age $t_j$ is calculated as the proportion of *Q* sampled permutations where the variance $T_j^{(q)}$ is greater than or equal to the observed variance $T_j$.

We remark that the integral of average network measure $I_k^{(q)}(t_j)$ provides a comprehensive perspective of the temporal evolution of SCNs from $t_j$ to $t_N$, reflecting the cumulative effects of sex and maternal education, and hence a reasonable quantity to consider for permutation tests. We avoid a multiple comparisons problem as we aim to test whether or not sex and maternal education affect the temporal evolution of SCNs after a specific age, rather than at all ages. Similar permutation tests can be applied to investigate the statistical significance of the relationship between cognitive development and the evolution of SCNs. Specifically, the ELC scores are randomly permuted *Q* times, where each time the integral of average network measure from $t_j$ to $t_N$ is calculated for each of the three ELC scores 80, 100, 120. The test statistic for the permutation test at age $t_j$ is then the variance of the resulting three integrals of average network measure.

### Statistical analysis overview

For sex and maternal education effects, local Fréchet regression was conducted for each of the four groups split by sex and maternal education (low or high), with the age at which the child was examined as the covariate. To assess the association between cognitive development and the evolution of brain region volumes, age and ELC score were included as covariates in local Fréchet regression, where no grouping was involved. For all analyses, all available samples were considered and the prediction was evaluated at ages varying from 1 to 9 to minimize boundary effects. Local Fréchet regression was implemented using the R package frechet^58^ (https://cran.r-project.org/web/packages/frechet/index.html). The R package igraph^59^ was used to calculate network measures. All visualizations are obtained using the R package ggplot2^60^.

## Supplementary Information

Supplementary Information.

## Data Availability

The datasets generated during and/or analyzed during the current study are available in the Dryad repository, https://doi.org/10.25338/B8B077.
