# Supplementary material for: Network evolution of regional brain volumes in young children reflects neurocognitive scores and mother’s education
Source: Sci Rep. 2023 Feb 20;13:2984. doi: 10.1038/s41598-023-29797-1 (PMC9941570; doi:10.1038/s41598-023-29797-1)
Supplement: Supplementary file 1 — Supplementary Information. [file 41598_2023_29797_MOESM1_ESM.pdf]

## Supplementary material

**Table S1.** Anatomical regions of interest (ROIs) for the DKT atlas. See also https://mindboggle.readthedocs.io/en/latest/labels.html.

| ROI | Description |
|---|---|
| Non-cortex | |
| 4 | left lateral ventricle |
| 6 | left cerebellum exterior |
| 7 | left cerebellum white matter |
| 10 | left thalamus proper |
| 11 | left caudate |
| 12 | left putamen |
| 13 | left pallidum |
| 14 | 3rd ventricle |
| 15 | 4th ventricle |
| 16 | brain stem |
| 17 | left hippocampus |
| 18 | left amygdala |
| 24 | CSF |
| 26 | left accumbens area |
| 28 | left ventral DC |
| 43 | right lateral ventricle |
| 45 | right cerebellum exterior |
| 46 | right cerebellum white matter |
| 49 | right thalamus proper |
| 50 | right caudate |
| 51 | right putamen |
| 52 | right pallidum |
| 53 | right hippocampus |
| 54 | right amygdala |
| 58 | right accumbens area |
| 60 | right ventral DC |
| 630 | cerebellar vermal lobules I-V |
| 631 | cerebellar vermal lobules VI-VII |
| 632 | cerebellar vermal lobules VIII-X |
| Cortex | |
| Left cortex | |
| 1002 | left caudal anterior cingulate |
| 1003 | left caudal middle frontal |
| 1005 | left cuneus |
| 1006 | left entorhinal |
| 1007 | left fusiform |
| 1008 | left inferior parietal |
| 1009 | left inferior temporal |
| 1010 | left isthmus cingulate |
| 1011 | left lateral occipital |
| 1012 | left lateral orbitofrontal |
| 1013 | left lingual |
| 1014 | left medial orbitofrontal |
| 1015 | left middle temporal |
| 1016 | left parahippocampal |
| 1017 | left paracentral |
| 1018 | left pars opercularis |
| 1019 | left pars orbitalis |
| 1020 | left pars triangularis |
| 1021 | left pericalcarine |
| 1022 | left postcentral |
| 1023 | left posterior cingulate |
| 1024 | left precentral |
| 1025 | left precuneus |
| 1026 | left rostral anterior cingulate |
| 1027 | left rostral middle frontal |
| 1028 | left superior frontal |
| 1029 | left superior parietal |
| 1030 | left superior temporal |
| 1031 | left supramarginal |
| 1034 | left transverse temporal |

| | |
|---|---|
| 1035 | left insula |
| Right cortex | |
| 2002 | right caudal anterior cingulate |
| 2003 | right caudal middle frontal |
| 2005 | right cuneus |
| 2006 | right entorhinal |
| 2007 | right fusiform |
| 2008 | right inferior parietal |
| 2009 | right inferior temporal |
| 2010 | right isthmus cingulate |
| 2011 | right lateral occipital |
| 2012 | right lateral orbitofrontal |
| 2013 | right lingual |
| 2014 | right medial orbitofrontal |
| 2015 | right middle temporal |
| 2016 | right parahippocampal |
| 2017 | right paracentral |
| 2018 | right pars opercularis |
| 2019 | right pars orbitalis |
| 2020 | right pars triangularis |
| 2021 | right pericalcarine |
| 2022 | right postcentral |
| 2023 | right posterior cingulate |
| 2024 | right precentral |
| 2025 | right precuneus |
| 2026 | right rostral anterior cingulate |
| 2027 | right rostral middle frontal |
| 2028 | right superior frontal |
| 2029 | right superior parietal |
| 2030 | right superior temporal |
| 2031 | right supramarginal |
| 2034 | right transverse temporal |
| 2035 | right insula |

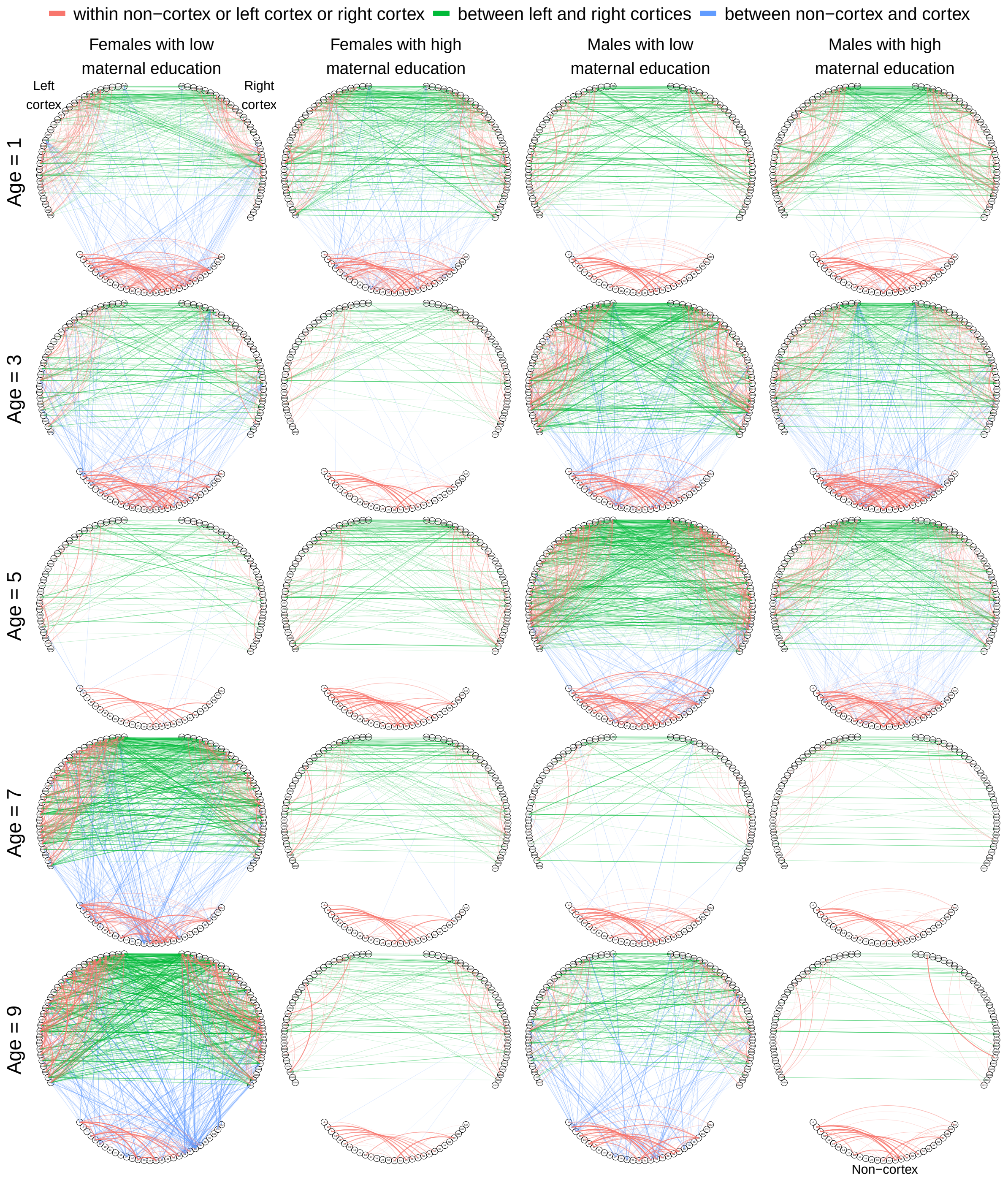


**Figure S1.** SCNs at ages 1, 3, 5, 7, 9 for the four groups divided by sex and maternal education using circle layout. Rows from top to bottom correspond to the five ages. Columns from left to right correspond to the four groups: females with low maternal education, females with high maternal education, males with low maternal education, males with high maternal education. The non-cortical regions are placed on the top. The left and right cortical regions are placed on the bottom left and right, respectively; see Table S1 for details of ROIs.

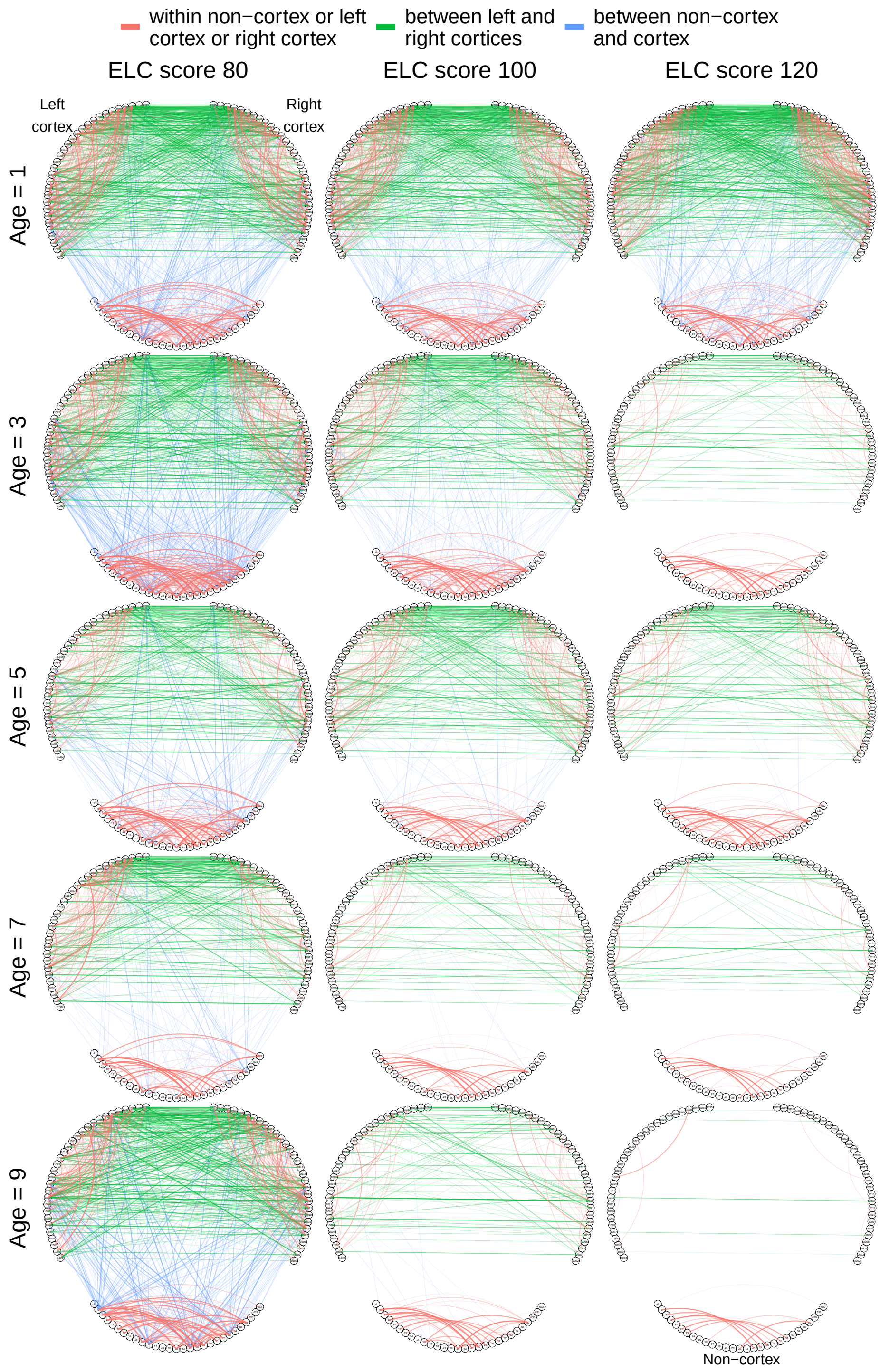


**Figure S2.** SCNs at ages 1, 3, 5, 7, 9 for ELC scores 80, 100, and 120 using circle layout. Rows from top to bottom correspond to the five ages. Columns from left to right correspond to the three ELC scores. The non-cortical regions are placed on the top. The left and right cortical regions are placed on the bottom left and right, respectively; see Table S1 for details of ROIs.
